# Evaluating the Safety of Ultra-Low-Dose Estrogen Contraception in Sickle Cell Trait With Focus on Cerebral Venous Sinus Thrombosis

**DOI:** 10.7759/cureus.34163

**Published:** 2023-01-24

**Authors:** Cameron Kahn, Azeem Rathore, Tara Kronen, Ameen Fahad, Ryan Crooks

**Affiliations:** 1 Medicine, University of Florida College of Medicine, Jacksonville, USA; 2 Internal Medicine, University of Florida College of Medicine, Jacksonville, USA; 3 Neurology, University of Florida College of Medicine, Jacksonville, USA

**Keywords:** ultra-low-dose estrogen, superior sagittal sinus thrombosis, loestrin, oral contraceptive pills, cerebral venous thrombosis, sickle cell trait

## Abstract

Ultra-low-dose combination estrogen-progestin contraceptive pills (OCP) have been marketed as being safer to use than previously higher estrogen-containing OCPs. While multiple large studies have shown a dose-dependent association between estrogen and deep vein thrombosis, there remains sparse guidance or data as to whether patients with sickle cell trait should avoid estrogen-containing OCPs regardless of the dosage. We present a case of a 22-year-old female with a history of sickle cell trait who had recently been started on an ultra-low-dose norethindrone-ethinyl estradiol-iron (1-20 mcg) that presented with headache, nausea, vomiting, and obtunded. Initial neuroimaging was significant for an extensive superior sagittal sinus thrombosis with extension into the confluence of dural venous sinuses, right transverse sinus, right sigmoid sinus, and right internal jugular vein which ultimately required systemic anti-coagulation. Her symptoms largely resolved within four days after starting anti-coagulation. She was discharged on day six to complete a six-month course of oral anti-coagulation. At her neurology follow-up three months later, the patient reported resolution of all symptoms. This study evaluates the safety of ultra-low-dose estrogen-containing contraceptive pills in the sickle cell trait population with special focus on cerebral sinus thrombosis.

## Introduction

It is well established that estrogen-containing oral contraceptive pills (OCPs) are linked to the risk of thrombosis. Despite data suggesting the elevated risk of deep vein thrombosis (DVT) and pulmonary embolism (PE) in the sickle cell trait population, there remains no cautionary labeling for OCP use in this population cohort. With the advent of ultra-low-dose estrogen-containing OCPs now available in the market, providers may be more inclined to prescribe OCPs to this at-risk population. One well-known complication related to OCP use is cerebral venous thrombosis (CVT) or cerebral venous sinus thrombosis (CVST). The use of OCPs has been shown in multiple observational studies to increase the odds of CVST by five-fold to 22-fold, however, no description of estrogen concentration has previously been documented [1]. In this study, we present a 22-year-old female with sickle cell trait who was recently prescribed an ultra-low-dose OCP for menorrhagia and subsequently developed extensive CVST less than 30 days later. Fortunately, she did not have further complications such as stroke or intracranial hemorrhage and tolerated systemic anti-coagulant therapy with complete resolution of her symptoms on outpatient follow-up. A literature review will be provided addressing the thrombotic risks associated with sickle cell trait patients and OCP use, in particular, ultra-low-dose estrogen pills followed by a brief discussion on CVST as it relates to ultra-low-dose estrogen.

## Case presentation

**Figure 1:**
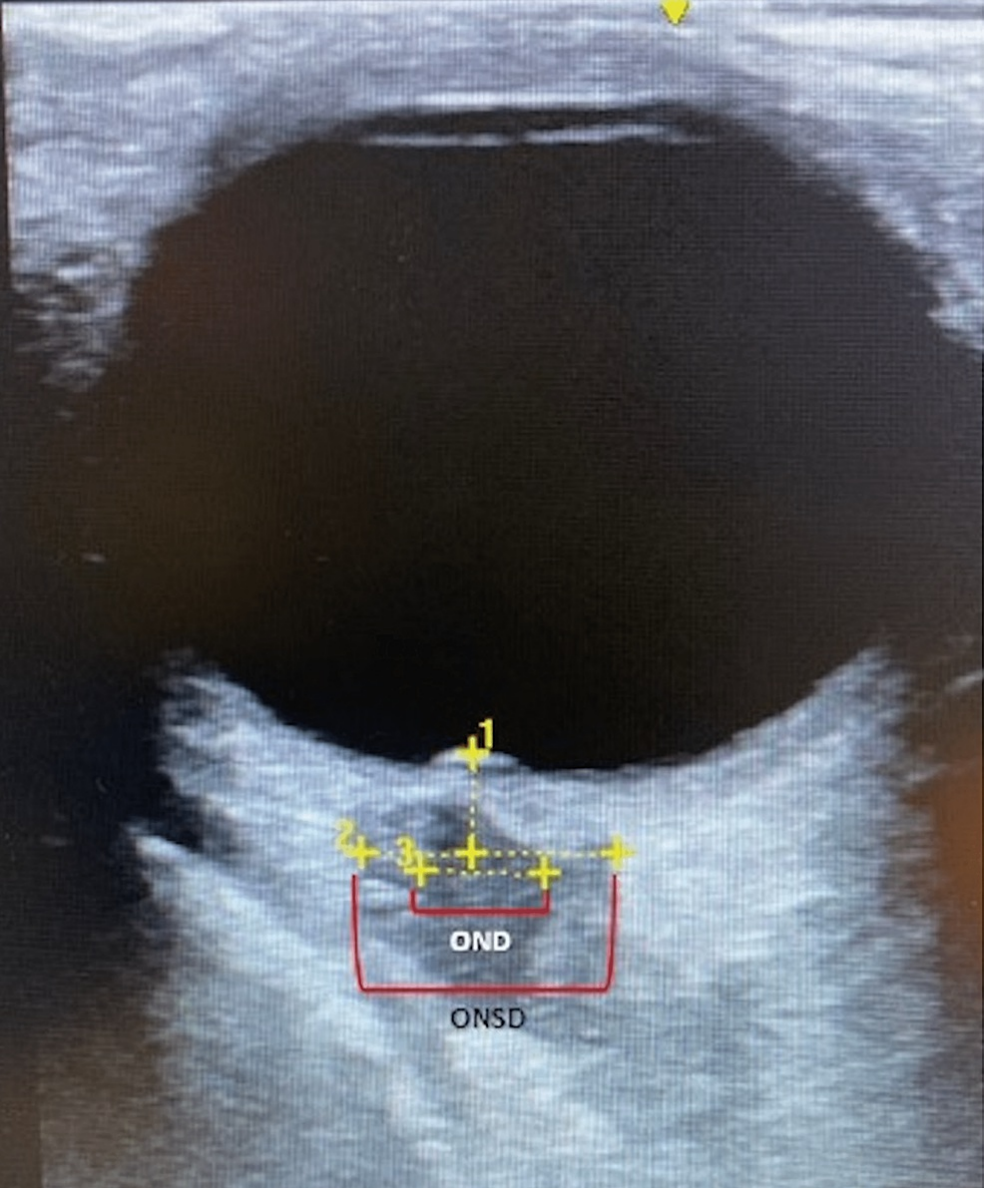
Right ocular ultrasonography in transverse plane. Papilla protrusion is shown which is suggestive of papilledema indicated by #1. A widened optic nerve sheath diameter (ONSD) of 7.6 mm is indicated by #2 and #3 shows the optic nerve diameter (OND). Both findings are suggestive of elevated intracranial pressure. Not pictured were the results of the ultrasonography of the left eye but were comparable in terms of widened optic nerve sheath diameter and papilledema.

A 22-year-old African-American female with a past medical history of sickle cell trait (unspecified), diabetes, morbid obesity, and menorrhagia presented with a chief complaint of worsening headache for three days. History was provided by the patient’s mother due to the patient’s transient altered mentation. The mother reported her daughter recently traveled out of town to visit family when she gradually developed a worsening headache along with weakness, unsteady gait, increased somnolence, neck stiffness, nausea, and non-bloody emesis. Her family noted her speech became less responsive being reduced to monosyllabic responses. The patient did deny fever, chills, rash, head injury, loss of consciousness, seizure activity, chest pain, evidence of bleeding, or exposure to wooded areas. History was negative for toxic ingestion or illicit substances. The patient was a nursing student, completing her clinical rotations in a local hospital living with several roommates in an apartment. She is up-to-date with all age-appropriate vaccinations and was recently started on norethindrone-ethinyl estradiol-iron 1-20 mcg (Loestrin-Fe®) OCP for her history of menorrhagia. On physical examination, blood pressure was 148/91 mmHg, heart rate was 96 beats/min, respiratory rate was 18 breaths/min, body temperature was 37.3°C, oxygen saturation was 100% on 3L nasal cannula, and her body mass index was 58 kg/m^2^. She appeared to be in moderate to severe distress, alert only to self but otherwise lethargic appearing that required a sternal rub to awaken her. Her Glasgow Coma Scale was 11 points. Neurological examination showed no focal deficits, though the assessment was incomplete as the patient could not follow all commands. Other pertinent findings included rotary nystagmus with light sensitivity, nuchal rigidity, and negative Kernig's and Brudzinski’s signs. No skin rashes were seen. Laboratory data revealed a mild leukocytosis of 19.94 x 10^9^/L and iron deficiency anemia with hemoglobin of 7.2 g/dL. A head computed tomography angiography did not show any intracranial hemorrhage or acute territorial infarct. Given the risk factors for idiopathic intracranial hypertension, an intraocular ultrasound was performed, which showed an optic nerve sheath diameter (ONSD) of 7.6 mm and papilledema, consistent with elevated intracranial pressure (ICP) (Figure 1). In addition, blood cultures and urine cultures returned negative.

**Figure 2:**
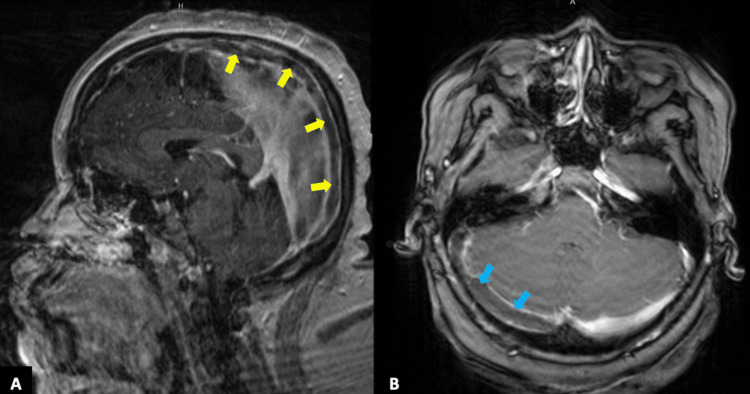
T1-weighted contrast-enhanced MRI of the brain. Sagittal reformatted image (A) demonstrating a filling defect within the superior sagittal signal (yellow arrows) consistent with thrombosis. The axial image (B) demonstrates the extent of the thrombosis with extension into the right transverse sinus (blue arrows).

**Figure 3:**
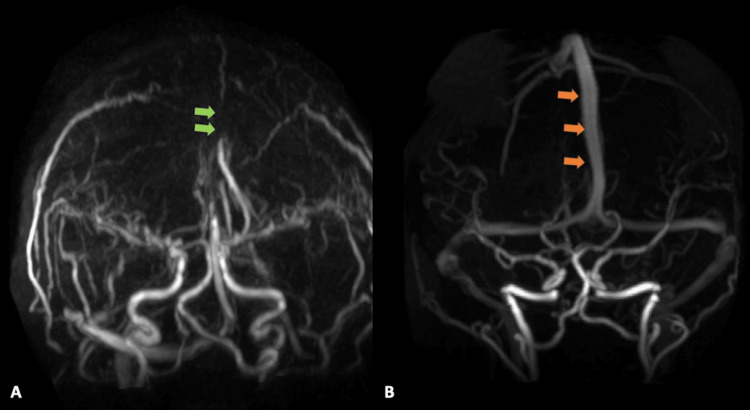
MR venography with contrast. The image in (A) demonstrates an absence of gadolinium contrast filling in the superior sagittal sinus (green arrows) compared to image (B) which shows a normal patent superior sagittal sinus (orange arrows).

A lumbar puncture revealed clear cerebral spinal fluid with 333 red blood cells/UL, 4 white blood cells/UL, glucose of 56 mg/dL, and an elevated protein of 58 mg/dL, along with a mildly elevated opening pressure of 27 cm of H_2_O and closing pressure of 19 cm of H_2_O. A single dose of acetazolamide was given to reduce intracranial pressure. Further, given her risk factors for meningitis, the patient was empirically started on acyclovir, cefepime, vancomycin, and dexamethasone. She was admitted to the internal medicine service for further management. Neurology was consulted and recommended a magnetic resonance venography (MRV) brain that revealed extensive thrombosis of the superior sagittal sinus from anterior to posterior with extension into the confluence of dural venous sinuses, right transverse sinus, right sigmoid sinus, and right internal jugular vein (Figures 2A, 2B, 3A, 3B).

The patient was diagnosed with extensive CVST, started on a therapeutic heparin infusion, and transferred to the neurology intensive care unit for further monitoring. Neurosurgery was consulted and recommended no surgical intervention. Further diagnostic work-up included a negative toxicology screen, an unremarkable hypercoagulable work-up (including an anti-nuclear antibody {ANA} titer, anti-double stranded DNA {anti-dsDNA}, lupus anti-coagulant, Factor V Leiden, and protein C/S deficiency). Her hemoglobin remained stable and she did not experience complications from the heparin infusion. Over the next four days, her mentation and symptoms improved as well. She was discharged on day six with apixaban 5 mg twice daily and instructed not to restart her OCPs until further discussion with her gynecologist. At her neurology follow-up three months later, the patient reported complete resolution of all symptoms.

## Discussion

Since its inception in the 1960s, OCPs have been the most common form of birth control in the United States. Over the past few decades, new formulations have been designed to reduce the dose of estrogen which has been linked to major side effects as previously elucidated. Labeled as "ultra-low-dose," newer OCP formulations have been dosed to 10-20 mcg compared to doses ranging up to 50 mcg. In theory, the lower estrogen doses should yield fewer side effects including fewer headaches, decreased gastrointestinal symptoms, less fluid retention, less bleeding, and lower clot risk while still maintaining similar efficacy in preventing pregnancy as seen with the higher doses [2]. Through the coagulation cascade, OCPs increase factors II, VII, VIII, X, and fibrinogen in the plasma, while also decreasing factor V, leading to increased clot formation [3,4]. Warning labels on the prescription package caution consumers and physicians from prescribing even the ultra-low-dose OCPs to patients with coagulopathies as their risk of thrombosis is still high. However, there is sparse reporting on whether individuals with sickle cell trait should also be considered at-risk. It is well documented that OCPs over 50 μg have a 10-fold increased risk of DVT when compared to non-users [5,6]. The dose effect has been established through numerous studies noting an inverse relationship between the risk of DVT to the estrogen dose with some citing a four-fold increased risk for DVT in users of OCPs that contained <50 μg and even lower with doses approaching 20-30 μg when compared to non-users [5,6]. Weill et al. showed in their population-based cohort study that levonorgestrel, with 20 µg of estrogen, was associated with a significantly lower risk of PE, ischemic stroke, and myocardial infarction than levonorgestrel with 30-40 µg of estrogen [7]. For the same type of progestogen, an estrogen dose of 20 µg compared to 30-40 µg was associated with a lower risk of PE (by 25%), ischemic stroke (by 18%), and myocardial infarction (by 44%) [7]. In the context of the aforementioned studies, the literature has been lacking in examples of OCP use in the sickle cell trait population. As it relates to our case, the patient was diagnosed with unspecified sickle cell trait and was experiencing mild menorrhagia for which her primary care physician started her on norethindrone-ethinyl estradiol-iron (1-20 mcg) (Loestrin Fe®) [8]. In our study, the patient was started on Loestrin Fe® and less than one month later she developed a DVT in her cerebral venous sinus. In light of conventional risk factors being ruled out, including a negative hypercoagulable work-up, no smoking history, and medication reconciliation that did not identify any pro-thrombotic medicines, it was strongly suspected her CVST was likely due to a combination of OCP use in the background of her known sickle cell trait.

Currently, there is sparse and conflicting data on whether sickle cell trait carrier increases the risk of venous thrombosis events. In one prospective population-based cohort investigation, sickle cell trait in African Americans carried a two-fold increased risk of PE but did not elevate DVT risk [9,10]. Alternatively, a cohort study with nested case-control analysis determined sickle cell carriers (sickle cell trait) remained at increased risk of venous thromboembolism (VTE) after adjusting for body mass index, pregnancy, smoking status, and ethnicity with the greatest risk for PE [11]. Further, one meta-analysis of sickle cell trait patients reported a higher risk of both VTE and PE but not DVT when compared to the control groups; of note, though, OCP use was not reported in their study [12]. There have been some studies evaluating the association between hormonal contraceptives and VTE risk in sickle cell trait cohorts. For example, one case-control study showed that the association between hormonal contraceptive use and VTE risk among African Americans was increased among women with sickle cell trait [13]. However, not only were the differences not statistically significant, the authors failed to discuss the doses of the hormonal contraceptive drugs included. The use of OCPs has been shown in several observational studies to increase the odds of CVST by five- to 22-fold, however, there has not been any explicit discussion of estrogen dosing [1]. Ibrahim et al. performed a retrospective case-control study to assess the relative risk of CVST among users on OCPs compared with a control group not using OCPs [14]. They found the risk of increased CVST more in the OCP users than in the non-hormonal users and further identified that the dural sinus, specifically the superior sagittal sinus, was the most implicated structure similar to our patient [14,15].

The three main risk factors for CVST are young age, female gender, and oral contraceptive use. The most common symptom is headache followed by vomiting [14]. Table 1 summarizes a complete list of predisposing conditions [15-17].

**Table 1:** Predisposing conditions to the development of cerebral venous sinus thrombosis (CVST). This is not an exhaustive list.

CVST predisposing conditions
Recent surgery
Prior medical conditions (thrombophilia, anti-phospholipid syndrome, cancer, inflammatory bowel disease)
Transient situations (pregnancy, puerperium, dehydration, infection, dehydration, infection)
Medications (oral contraceptives, exogenous hormones, substance abuse)
Unpredictable events (head trauma)

Patients can present with CVST in a number of clinical syndromes including idiopathic intracranial hypertension (IIH), encephalopathy, or more focal neurologic syndromes [18-20]. In our patient, the differential diagnosis was initially broad. Due to her recent travel to Virginia, occupational exposure, and close-quarters living situation, we initiated empiric coverage for meningitis and prophylactically administered acetazolamide for IIH especially given her young age, morbid obesity, and ultrasound findings consistent with elevated ICP. Finally, in the context of her OCP use, history of menorrhagia, and sickle cell trait, we considered the presence of a cerebral thrombosis versus subacute ischemic stroke and additional neuroimaging ultimately confirmed the presence of CVST.

There are two concomitant hypothesized mechanisms involved in the deleterious effects of CVST. The occlusion of the cerebral vein leading to localized edema and venous ischemia, and the occlusion of major venous sinuses leading to impaired absorption of cerebrospinal fluid, thus increasing intracranial hypertension [21]. It is theorized that patients with sickle cell and sickle cell trait develop venous sinus thrombosis due to venous stasis from increased blood viscosity during hypoxic episodes [22]. Despite this theory, sickle cell trait as a risk factor for CVST is rarely reported especially if proper neuroimaging is not done. Indeed, MRI and MRV are the most commonly used, but MRI imaging of these patients will actually show normal parenchyma [14]. In our patient, her MRI brain had normal parenchyma without associated infarction but the filling defect within the dural venous sinus was clearly observed with follow-up MRV noting absent filling within the superior sagittal sinus and right transverse sinus.

The cornerstone of CVST management involves systemic anti-coagulation. Adjunct therapy, such as acetazolamide to reduce intracranial pressures secondary to clot burden, is commonly used despite the lack of randomized control trials. Canhão et al.performed a prospective observational study on the role of steroids in cerebral venous thrombosis patients’ and found no benefit [23]. Serial lumbar punctures may be needed if no improvement to neurologic status is seen despite anti-coagulation and we propose monitoring intracranial pressures via ocular ultrasound to help guide the decision to repeat a lumbar puncture if clinical status remains unchanged [24]. No repeat lumbar puncture was performed in our patient as she showed clinical improvement after the initiation of anti-coagulation. In some cases where anti-coagulation is insufficient, lumboperitoneal shunting may be indicated while in cases of CVST complicated by cerebral hemorrhage mechanical thrombectomy followed by local thrombolytics would be indicated [25]. Finally, it is important to note that the CVST mortality rates at 30 days and one year are 3% and 6%, respectively [26]. For our patient who was discharged six days after admission at her follow-up visits at week 1 and week 3, she continued to endorse occasional photophobia and headaches but at month three her symptoms had completely resolved.

## Conclusions

This study highlights how sickle cell trait patients may be an at-risk population of developing VTE while using OCP, especially with the advent of ultra-low-dose combination estrogen-progestin contraceptive pills. While a cause-effect relationship cannot be determined due to the nature of case reports, clinicians should consider the potential VTE risk of ultra-low-dose OCP in sickle cell trait patients. Ultimately, further research is required to understand if a dose-effect relationship exists between estrogen concentrations of OCPs and the development of cerebral venous sinus thrombosis (CVST), namely in the form of large cohort studies.
